# Interleukin-17/β-Defensin-2 Expression Before and After Periodontal Therapy in a Patient With Periodontitis/Autoimmunity

**DOI:** 10.7759/cureus.78657

**Published:** 2025-02-06

**Authors:** Saira Karina Ramírez-Thomé, Risk Díaz-Castillejos, César Eslí Rabadán-Martínez, Roberta Lizette Palacios-Cruz, Eunice Daysi García-Reyes, César Zárate-Ortíz, Beatriz Xóchitl Ávila-Curiel, Edgar Zenteno-Galindo, Carlos Josué Solórzano-Mata

**Affiliations:** 1 Faculty of Dentistry, Universidad Autónoma Benito Juárez de Oaxaca, Oaxaca, MEX; 2 Faculty of Nursing, Universidad Autónoma Benito Juárez de Oaxaca, Oaxaca, MEX; 3 Faculty of Medicine and Surgery, Universidad Autónoma Benito Juárez de Oaxaca, Oaxaca, MEX; 4 Faculty of Medicine, Universidad Nacional Autónoma de México, Mexico City, MEX

**Keywords:** interleukine-17, periodontitis, psoriasis, rheumatoid arthritis, β-defensin-2

## Abstract

Periodontitis is a chronic inflammatory disease that affects the supporting tissues of the periodontium. Comorbidity in patients with periodontitis with rheumatoid arthritis and psoriasis has been documented. In immunopathogenesis, it has been described that interleukin 17 participates in periodontitis and autoimmune diseases. In this regard, interleukin 17 induces the expression of β-defensin-2, an antimicrobial peptide with a proinflammatory effect. Currently, it is not known how periodontal therapy impacts the production of interleukin 17 and β-defensin-2. This is the first case report exploring the modification of interleukin 17/β-defensin-2 in a patient with periodontitis or autoimmunity, showing the importance and effect of non-surgical therapy on modifications of the immune response at the molecular level. The aim of this work is to evaluate the clinical improvement and the levels of interleukin 17/β-defensin-2 in saliva before and after periodontal treatment in a patient with periodontitis, rheumatoid arthritis, and psoriasis. We present the case of a 51-year-old male patient with a history of psoriasis and rheumatoid arthritis. On periodontal examination, he presented with periodontal pockets greater than 5 mm in 19 periodontal sites, with 100% bleeding on probing, and tooth mobility grades II and III in most of the dental organ. Subgingival instrumentation and oral hygiene instructions were performed. The patient showed favorable progress, with a reduction in periodontal pocket depth, bleeding on probing, and tooth mobility. Interleukin 17 levels were higher and β-defensin-2 levels were lower post-treatment. Non-surgical treatment decreased the inflammatory activity of periodontal disease, which led to increased interleukin 17 and decreased β-defensin-2 in saliva samples. The decrease in β-defensin-2 suggests its usefulness as a marker of inflammatory activity in periodontal disease.

## Introduction

Periodontitis (Per) is a multifactorial chronic inflammatory disease associated with dysbiotic plaque biofilms, characterized by progressive destruction of the supporting apparatus of the periodontium [1]. In addition, it has been associated with other diseases, such as rheumatoid arthritis (RA) and type 1 diabetes mellitus (DM-I) [2].

Gingival mucosal epithelial cells secrete antimicrobial peptides (AMPs) such as β-defensin-2 (hBD-2), which has been confirmed both by the identification of its mRNA in these cells and by the presence of the peptide itself in saliva [3]. At the tissue level, gingival biopsies have demonstrated that hBD-2 is found at a very low level in the gingival tissue of healthy individuals with no signs of inflammation [3]. In contrast, the production and secretion of hBD-2 by oral keratinocytes are induced following stimulation by proinflammatory cytokines such as tumor necrosis factor-α (TNF-α), interleukin-1β (IL-1β), interferon-γ (IFN-γ), and interleukin-17 (IL-17), or as a result of bacterial endotoxins, such as lipopolysaccharide (LPS) from *Fusobacterium nucleatum* (*F. nucleatum*) and *Aggregatibacter actinomycetemcomitans* (*A. actinomycetemcomitans*) [3].

IL-17 is a proinflammatory cytokine produced by Th17 lymphocytes, Tγδ lymphocytes, lymphoid tissue-inducing cells (LTi), innate lymphoid cell type 3 (ILC3), and natural killer (NK) cells [4]. Interestingly, IL-17 stimulates epithelial cells to secrete hBD-2 upon binding of IL-17A to the IL-17RA receptor. At the pathological level, IL-17 is involved in the pathogenesis of allergy, malignancies, and autoimmune diseases such as RA, systemic lupus erythematosus (SLE), multiple sclerosis, psoriasis, and Per [4].

At the periodontal level, the increase of IL-17 in the context of Per is accompanied by an increase in microbial load and the presence of dysbiotic bacterial communities. In fact, it is considered a critical component of periodontal inflammation [5]. Likewise, the proportion of Th17/Treg cells in gingival tissue and peripheral blood of patients with Per was significantly higher than in healthy individuals, and the number of Tγδ cells in gingival tissue was also higher than in healthy individuals [6]. Both Th17 and Tγδ cells are the main cellular sources of IL-17A, suggesting that the level of IL-17A expression in the periodontal region of patients with Per is significantly higher than that of healthy individuals [6].

In this regard, a correlation has been established between RA-associated Per and *Porphyromonas gingivalis* (*P. gingivalis*). So far, *P. gingivalis* is the only bacterium known to express the enzyme peptidyl arginine deiminase (PAD) [7], which is responsible for performing post-translational protein modifications similar to those carried out by human PAD. Although PAD expressed in *P. gingivalis* differs from the human variant, it has been shown that it can produce irreversible citrullinated peptides from at least two known RA antigens: fibrinogen and α-enolase. This suggests that *P. gingivalis* is associated with an increased risk of developing RA [7].

In addition, PAD may act in concert with *P. gingivalis* arginine-specific proteinases, promoting the growth of the pathogen in the periodontal pocket by enhancing its ability to survive and subsequently helping the organism evade the host's humoral defenses [7].

RA is a chronic autoimmune inflammatory disease that affects the synovial membranes in multiple joints, causing inflammation and eventually tissue destruction [8]. In patients with RA, PAD adds citrulline to arginine residues in host proteins, mediated by the PAD family, which are not recognized as self by the immune system, leading to the formation of anti-citrullinated protein antibodies, with consequent activation of the immune response and tissue damage [8].

Interestingly, an association between RA, Per, and tooth loss has been reported. In this regard, a connection has been established between periodontal pathogens such as *P. gingivalis* and *A. actinomycetemcomitans* in the citrullination process, leading to the formation of autoantibodies and compromised immunotolerance in RA-susceptible patients [9].

In saliva samples of RA patients, the level of IL-17A was found to be higher compared to healthy controls but lower than that of Per patients. Meanwhile, the level of IL-17A in the saliva of RA patients treated with antirheumatic drugs was reduced [10]. Moreover, serum samples from RA and Per patients showed that the concentration of IL-17A was significantly higher than that of Per patients without systemic diseases [10]. These data suggest that the dysbiotic oral microbiota in Per may play a role in the development or exacerbation of RA, as it may influence IL-17 production and consequently promote the immune response by favoring the inflammatory phenomenon [10].

Psoriasis is an autoimmune disease of the skin, characterized by the proliferation of keratinocytes that form hyperkeratinized squamous plaques [11]. Although its pathobiological mechanisms remain unclear, it has been proposed that abnormal activation of the innate and adaptive immune systems causes characteristic local inflammatory reactions in the skin, as well as low-grade systemic inflammation with elevated circulating levels of inflammatory cytokines [11]. Cross-sectional studies and systematic reviews demonstrate a higher prevalence and increased activity of Per in patients with psoriasis compared to periodontally healthy controls [11].

In patients with psoriasis and moderate or severe Per, total concentrations of IL-17A, IL-22, and IL-23 in the gingival crevicular fluid were found to be increased compared to patients with psoriasis and mild or no Per [11]. It has even been reported that a bidirectional relationship exists between Per and psoriasis, where one disease increases the risk of the other [11].

Considering the common characteristics of Per, RA, and psoriasis, IL-17 can be identified as a key and shared cytokine linking these three conditions. As previously described, there is a significant increase in IL-17 expression in diverse lesions within the oral cavity, joints, and skin.

A possible immunopathogenic mechanism may involve the loss of tolerance to citrullinated proteins induced by *P. gingivalis*, an event that favors autoimmunity and systemic proinflammatory activity. In addition to its establishment in periodontal pockets, this leads to the destruction of periodontal tissues, which has been associated with chronic inflammation and sustained increases in IL-17 levels. Consequently, this could contribute to the onset of Per, joint destruction, and the appearance of skin lesions. Therefore, it has been suggested that Per plays a role in the development and progression of RA and psoriasis [12].

Considering the available evidence, little is known about the changes that may occur in IL-17 and hBD-2 following periodontal treatment in patients with Per, RA, and psoriasis. The aim of this work is to evaluate IL-17 and hBD-2 levels in saliva before and after periodontal treatment in a patient with Per, RA, and psoriasis.

## Case presentation

**Figure 1:**
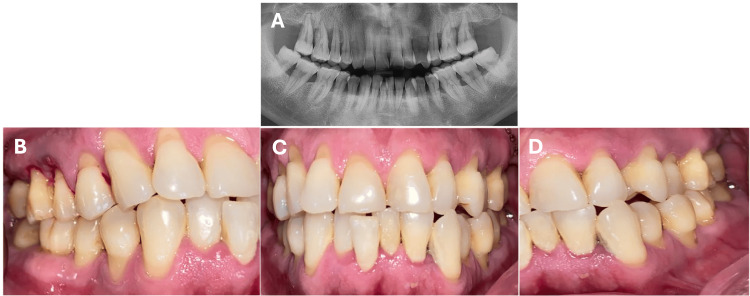
Initial clinical presentation of the patient diagnosed with stage IV, grade C, generalized periodontitis. (A) Panoramic radiograph taken prior to the start of periodontal treatment, showing severe generalized bone loss around the teeth. (B) Right lateral photograph, (C) left lateral photograph, and (D) frontal view showing generalized inflamed periodontal tissues, with multiple gingival recessions, gingival bleeding, dental crowding, the presence of dental calculus, and gingival erythema.

A 51-year-old male presented to a private clinic in 2021 with a history of psoriasis and RA, under medical treatment with leflunomide. The patient is a chronic smoker, with a daily consumption of 10 cigarettes over the past 10 years. He presented for consultation due to gingival inflammation, bleeding, and tooth mobility. On periodontal examination, he exhibited periodontal pockets greater than 5 mm in 19 dental organs (DO), with 100% bleeding on probing (SS) and grade II and III tooth mobility in most of the DO radiographic evaluation revealed generalized bone loss of more than 50% (Figure 1).

Laboratory studies reflected adequate control of the rheumatoid factor (20 IU/mL, with a maximum reference value of 30 IU/mL). The red and white blood cell counts were within reference values. However, liver function tests showed elevated pyruvic transaminase at 46.7 U/L, considering a maximum reference value of 41.0 U/L, as well as C-reactive protein at 6.3 mg/L, compared to a maximum reference value of 6.0 mg/L (Table 1).

**Table 1:** Laboratory test results.

Laboratory parameter	Results	Normal values
Rheumatic factor (IU/mL)	20	<30
Pyruvic transaminase (U/L)	46.7	<41.0
C-reactive protein (mg/L)	6.3	<6.0

**Figure 2:**
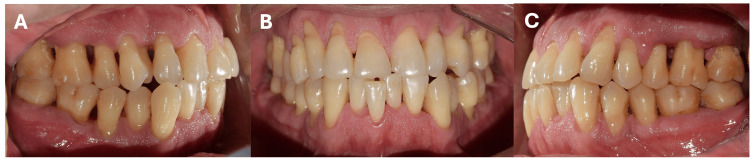
Clinical presentation after non-surgical periodontal treatment. (A) Right, (B) left, and (C) frontal photographs showing periodontal tissues without inflammation, no bleeding on probing, and the presence of interdental spaces as a consequence of attachment loss.

The diagnosis was stage IV periodontitis, grade C, generalized. Periodontal treatment consisted of subgingival instrumentation in all periodontal pockets and oral hygiene instructions. The treatment began with an initial appointment for calculus removal using ultrasonic instrumentation and dental polishing, along with oral hygiene instructions. This was followed by full-mouth scaling and root planing in two sessions within one week, using both ultrasonic and manual instrumentation under local anesthesia. All periodontal treatments and evaluations were performed by a single periodontist. The patient was prescribed metronidazole 500 mg every eight hours for seven days, and a follow-up appointment was scheduled one month later for reevaluation. At reevaluation, a reduction in periodontal pocket depth, a decrease in the percentage of SS, and reduced dental mobility were observed (Figure 2).

On the other hand, two saliva samples were collected: the first before initiating periodontal treatment and the second at the periodontal reevaluation, in order to quantify the levels of IL-17 and hBD-2.

Regarding IL-17 levels, these were lower before treatment compared to post-treatment levels and those of the control from a periodontally healthy individual. For hBD-2 concentrations, the levels were higher before treatment compared to post-treatment quantification, as well as the control (Table 2). 

**Table 2:** Quantification of IL-17 and hBD-2 in total saliva in patient with Per, RA, and psoriasis, before and after periodontal therapy compared to a periodontal and systemically healthy subject.

Conditions	IL-17 (pg/mL)	hBD-2 (ng/dL)
Pre-treatment	40	411.95
Post-treatment	105.62	322.48
Periodontal and systemically healthy subject	96.36	96.064

## Discussion

The gold standard for the treatment of periodontal disease is patient education and motivation, along with non-surgical periodontal therapy, which involves subgingival instrumentation to modify bacterial biofilms [12]. Although the generalizability of the results is limited because this is a clinical case study, this work allows us to evaluate the behavior of hBD-2/IL-17 and explore possible molecular mechanisms. It has been documented that successful treatment of periodontal infection through non-surgical periodontal therapy has been associated with proper maintenance of RA and a reduction in the severity of active RA, confirming the role of Per not only in the onset but also in the progression of the disease [13]. In this case, periodontal disease activity improved dramatically, as indicated by a reduction in pocket depth and SS compared to the clinical findings observed prior to periodontal treatment.

On the other hand, Per and psoriasis share some immunopathological mechanisms, particularly their relationship to the IL-23/Th17/IL-17 axis [11]. One theory postulates that the underlying cause of inflammation at distant sites, such as the skin and joints, is due to an altered systemic immune balance caused by oral pathogens [11]. IL-17 is present in the immunopathogenesis of both Per and RA; however, our results are particularly interesting since IL-17 increased after treatment, a finding that aligns with other studies [14]. In this sense, IL-17A expression has been described as inversely proportional to the course and chronicity of RA, as IL-17 is mainly expressed in the preclinical phase and decreases with disease chronicity. This suggests that IL-17 levels may be regulated by various cytokines within the inflammatory environment and that it may exert an inhibitory effect on hBD-2, thereby decreasing its concentration after non-surgical periodontal treatment and reducing its potential negative effect on IL-17 [14].

In our study, we found that hBD-2 concentration was higher pre-treatment compared to post-periodontal treatment, a finding consistent with those reported in the literature in patients with psoriasis and RA, in whom a substantial increase in hBD-2 production has been observed compared to healthy controls. The production of hBD-2 by skin keratinocytes is positively regulated by inflammatory cytokines such as TNF-α, IFN-γ, IL-17, IL-12, IL-1β, and IL-6. In turn, hBD-2 in patients with psoriasis or RA increases the production of TNF-α, IFN-γ, IL-10, IL-1β, IL-6, and IL-22 [15].

**Figure 3:**
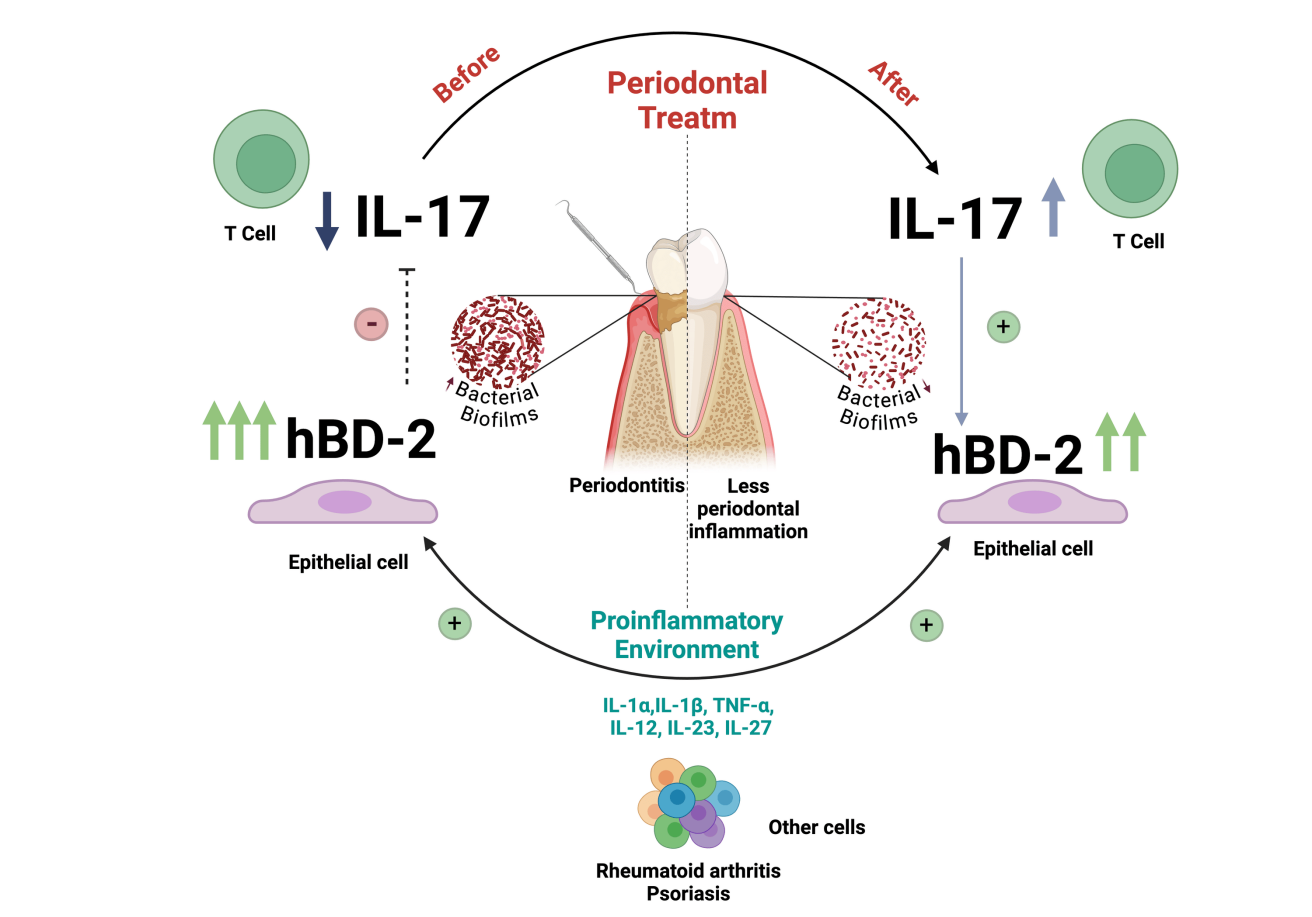
Effect of non-surgical periodontal therapy in a patient with periodontitis, rheumatoid arthritis, and psoriasis on the concentration of IL-17 and hBD-2 in unstimulated total saliva. The gold standard for the treatment of periodontal disease includes non-surgical periodontal therapy, consisting of subgingival instrumentation aimed at reducing bacterial biofilm, thereby decreasing inflammation and modifying the levels of soluble mediators of the immune response. In our patient, before periodontal treatment, the large amount of bacterial biofilm and other proinflammatory cytokines (favored by chronic autoimmune comorbidities) could have induced high concentrations of hBD-2, which may have exerted a negative effect on IL-17 levels. After periodontal therapy, the reduction in bacterial biofilm clinically decreased inflammation and increased IL-17 levels. This is likely due to the diminished negative effect induced by hBD-2 and other cytokines. Additionally, in this scenario, IL-17 may have played a favorable role in regulating hBD-2 concentrations. Image created by the authors using BioRender.com.

At this point, it is important to mention the possible regulatory interaction between IL-17 and hBD-2, where elevated levels of hBD-2 could have a negative effect on IL-17. This phenomenon has been described in T cells and may partly explain the increase in IL-17 levels after treatment in our patient, as it coincided with a decrease in hBD-2 concentrations and, consequently, its effect on IL-17 production (Figure 3) [15]. With respect to hBD-2 concentration, the positive regulatory role exerted by IL-17 on hBD-2 has been demonstrated; for example, high levels of hBD-2 correlate with low IL-17A levels and increased severity of psoriasis and/or RA [16]. In our patient, although there was a decrease in hBD-2 following periodontal treatment, it did not reach the concentrations observed in a healthy individual, suggesting that other cytokines may be involved in the positive regulation of hBD-2 and continue to be present despite periodontal and pharmacological treatment.

In the case presented, non-surgical treatment decreased periodontal disease activity, which impacted the increase of IL-17 and the decrease of hBD-2 in saliva samples.

Considering the results presented, we suggest conducting a similar study with a larger number of patients, as hBD-2 levels could be considered indicators of the inflammatory activity of periodontal disease [17]. In such a study, the identification and quantification of bacteria in total saliva before and after periodontal therapy should be included. In addition, the regulatory mechanisms between IL-17 and hBD-2 should be clarified, particularly the role of intermediary cytokines. For example, IL-17 can inhibit Th17 cells by inducing the production of IL-24, which, in turn, suppresses the production of IL-17, GM-CSF, and IL-22 in these cells, ultimately reducing stimulation for hBD-2 production [18]. Therefore, in the context of periodontal disease and autoimmunity, this mechanism should be further investigated.

## Conclusions

In conclusion, our results indicated that short-term non-surgical periodontal therapy led to a significant improvement in periodontal indices, which could have a potential impact on autoimmune diseases. This is partly suggested by the decrease in hBD-2 after therapy and the potential usefulness of this AMP as an indicator of inflammatory activity in periodontal disease among patients with RA and psoriasis.
